# Using electrical impedance tomography to estimate tidal volume in bottlenose dolphins and cape fur seals in seawater and on land

**DOI:** 10.1242/jeb.251412

**Published:** 2026-02-26

**Authors:** Andreas Fahlman, Randall S. Wells, Nicole West, Austin Allen, Andres Jabois, Tamaryn Gallagher, Josefin Larsson, Elin Strom, Martina Mosing, Tarek Harake, Andy Adler

**Affiliations:** ^1^ Linköping University, 581 83 Linköping, Sweden; ^2^ Fundación Oceanogràfic, 46013 Valencia, Spain; ^3^ Global Diving Research SL, 46004 Valencia, Spain; ^4^ Sarasota Dolphin Research Program, Brookfield Zoo Chicago, c/o Mote Marine Laboratory, Sarasota, FL 34236 USA; ^5^ Dolphin Quest, Honolulu, HI 96816, USA; ^6^ Atlantis, The Palm, Dubai, United Arab Emirates; ^7^ Kolmården Wildlife Park, 618 92 Kolmården, Sweden; ^8^ Clinical Centre for Small Animal Health and Research, Clinical Department for Small Animals and Horses, University of Veterinary Medicine Vienna, Veterinärplatz 1, 1210 Wien, Austria; ^9^ tnh Biosystems Inc., Ottawa, Canada; ^10^ Carleton University, Ottawa, ON K1V 8C6, Canada

**Keywords:** Spirometry, Respiratory monitoring, Marine mammal, Cetacean, Pinniped

## Abstract

Marine mammals possess specialized respiratory adaptations that enable efficient gas exchange and resilience to extreme pressures during diving, yet direct observation of lung mechanics under pressure has been logistically challenging. Electrical impedance tomography (EIT) measures real-time changes in thoracic impedance, and provides continuous, regional maps of pulmonary air distribution. We validated EIT for estimating tidal volume (*V*_T_) in bottlenose dolphins (*Tursiops* spp.) and Cape fur seals (*Arctocephalus pusillus*) both on land and in water. EIT reliably tracked *V*_T_ in both taxa, showing strong within-trial consistency, with between-trial variability attributable to belt placement, body position and electrode contact. EIT also generated dynamic functional images of regional ventilation, revealing spatial and temporal patterns of lung filling and emptying. These results demonstrate that EIT is the first non-invasive imaging method validated for marine mammals in seawater, representing a critical step toward visualizing lung function during diving.

## Teaser

**Summary:** A validation of electrical impedance tomography (EIT) for marine mammals demonstrates its first successful use in seawater, a major step toward underwater physiological applications.

## INTRODUCTION

Marine mammals, including odontocetes and pinnipeds, possess highly specialized respiratory systems that enable them to meet the extreme demands of life at sea (Scholander, 1940; Piscitelli et al., 2013; Fahlman, 2024; Piscitelli-Doshkov et al., 2024). Unlike terrestrial mammals, they must manage two seemingly conflicting physiological requirements: efficient gas exchange at the surface and resilience to the hydrostatic pressures experienced during breath-hold dives. Over millions of years of evolution, these animals have developed remarkable adaptations to meet these challenges. These include rapid, high-volume ventilation between dives, highly flexible thoracic structures, and physiological mechanisms that promote alveolar collapse at depth and reinflation at the surface (Fahlman, 2024; Fahlman et al., 2017; Piscitelli-Doshkov et al., 2024; Piscitelli et al., 2013; Scholander, 1940). Together, these traits form the foundation for their extraordinary diving capabilities.

Despite the central role that the lungs play in diving performance and survival, direct measurements of pulmonary function in marine mammals remain surprisingly limited. Much of what is known about lung compression, gas redistribution and alveolar collapse under pressure is inferred rather than observed (Falke et al., 1985; McDonald and Ponganis, 2012; Ridgway and Howard, 1979; Ridgway et al., 1969). Indirect approaches have included imaging cadavers, tracking changes in blood gases at depth, and modeling approaches (Bostrom et al., 2008; Fahlman et al., 2009, 2011, 2006; Falke et al., 1985; Kooyman et al., 1970, 1972; Kooyman and Sinnett, 1982; McDonald and Ponganis, 2012; Moore et al., 2011; Ridgway and Howard, 1979; Ridgway et al., 1969). Although invaluable, these methods offer only partial insight into the dynamic changes that occur in the lungs during breathing and diving. Until now, no approach has been able to capture these processes continuously across environments, on land and in water, in real time.

Electrical impedance tomography (EIT) represents a promising solution to this challenge. EIT is a non-invasive imaging technique that measures real-time changes in lung volume and the distribution of ventilation (Adler and Holder, 2022). Originally developed for human medicine, EIT is now used widely as both a clinical research tool and a diagnostic instrument (Adler and Holder, 2022). Its utility has also expanded into veterinary medicine, with successful applications in a diverse range of species, including horses, pigs, dogs, lambs, sheep, rhinoceroses and orangutans (Brabant et al., 2022; Mosing and Moens, 2022). It has been used to estimate tidal volume (*V*_T_) in horses and pigs (Crivellari et al., 2021; Mosing et al., 2022). As EIT is recorded in arbitrary units and does not reflect absolute lung volumes, a calibration step is necessary. A method for calibrating EIT involves aligning it with simultaneously recorded spirometry to derive a calibration slope, which is the linear relationship between impedance change (Δ*Z*) and *V*_T_. This slope acts as a scaling factor to convert EIT signals into volume-equivalent units, enabling quantitative assessment of lung function. Once established from a reference breath, the slope can be applied across subsequent breaths (Byrne et al., 2023).

Building on this foundation, we have adapted EIT for marine mammal research, allowing data collection in both terrestrial and aquatic settings (Adler et al., 2025). These developments represent an early step toward applying EIT to address longstanding questions in diving physiology, such as how lung mechanics adjust to changes in pressure and how these adaptations support the exceptional breath-hold capacities of marine mammals (Fahlman, 2024; Piscitelli-Doshkov et al., 2024). However, use during free-swimming behavior remains beyond current technical limits. Nonetheless, the ability to obtain reliable EIT signals in seawater represents a decisive breakthrough, establishing for the first time that this imaging technique can function in the conductive marine environment that had long prevented such measurements.

Given the promise of EIT, our goal was to validate its accuracy and robustness for estimating *V*_T_ in marine mammals in water. To accomplish this, we conducted a comparative study using paired EIT and ultrasonic spirometry measurements, the current gold standard, in bottlenose dolphins (*Tursiops* spp.) and Cape fur seals (*Arctocephalus pusillus*), representing contrasting diving ecologies. Trials were conducted in both terrestrial and aquatic environments to test the stability of EIT calibration factors across contexts.

We hypothesized that: (1) EIT-derived measurements would accurately track *V*_T_ compared with spirometry, and (2) calibration factors would remain consistent across environments, breathing phases and individuals. Our findings supported both hypotheses under controlled conditions in inactive animals: EIT captured *V*_T_ dynamics in both taxa, with calibration factors showing high stability within trials. These results support EIT as a useful, non-invasive tool for studying respiratory physiology in marine mammals in managed care or during brief capture–release assessment in wild animals.

## MATERIALS AND METHODS

All procedures were approved by the Animal Care and Welfare Committee of the Fundación Oceanogràfic (OCE-18-22-25), and the US Navy Bureau of Medicine and Surgery (BUMED, NRD no. 1286). The research on wild dolphins in Sarasota Bay, FL, USA, was conducted under a research permit issued by the National Marine Fisheries Service (Scientific Research Permit No. 26622).

**Fig. 1.**
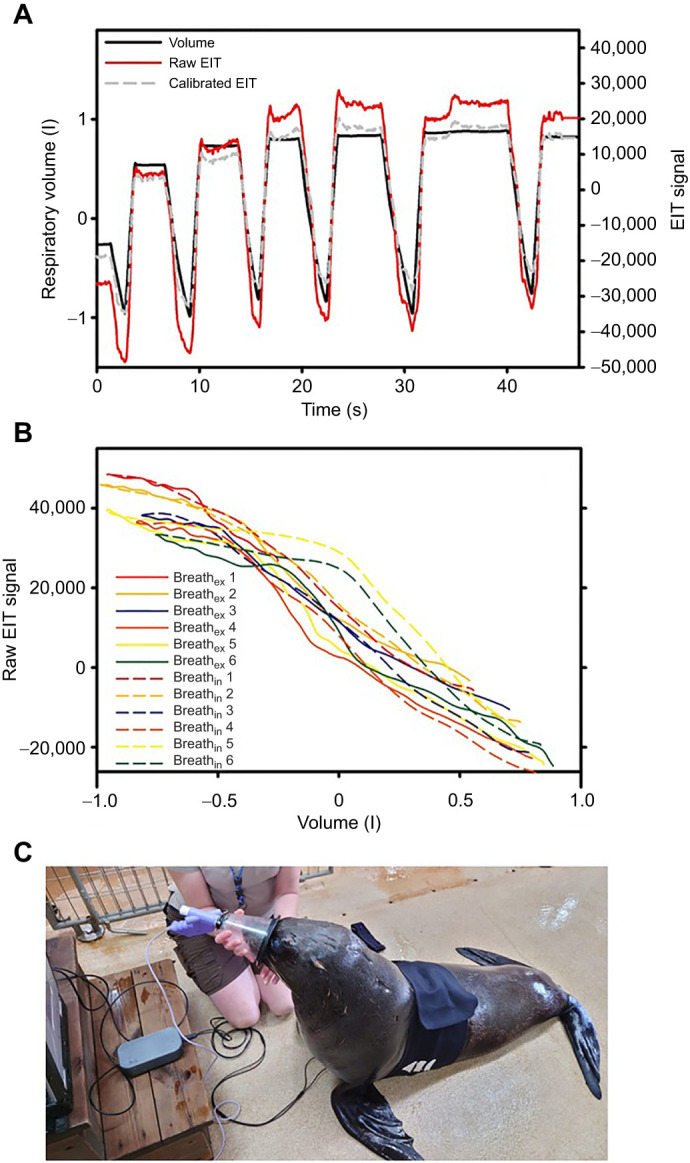
**Representative trial with six breaths from a Cape fur seal (*Arctocephalus pusillus*) showing how the data were analyzed.** (A) Respiratory volume corrected to standard temperature pressure dry (l STPD, black solid line), raw electrical impedance tomography (EIT) signal (red solid line; arbitary units) and calibrated EIT signal (l STPD, gray dashed line). For respiratory volume, downward and upward signals are, respectively, exhalation and inhalation. (B) Correlation between respiratory volume (l STPD) and raw EIT signal for each of the six breaths separated by exhalation (solid lines) and inhalation (dashed lines). The relationship for each breath was used to calibrate the EIT signal, which is plotted as a broken gray line in A. (C) Cape fur seal wearing the EIT belt and spirometer over the snout.

**Fig. 2.**
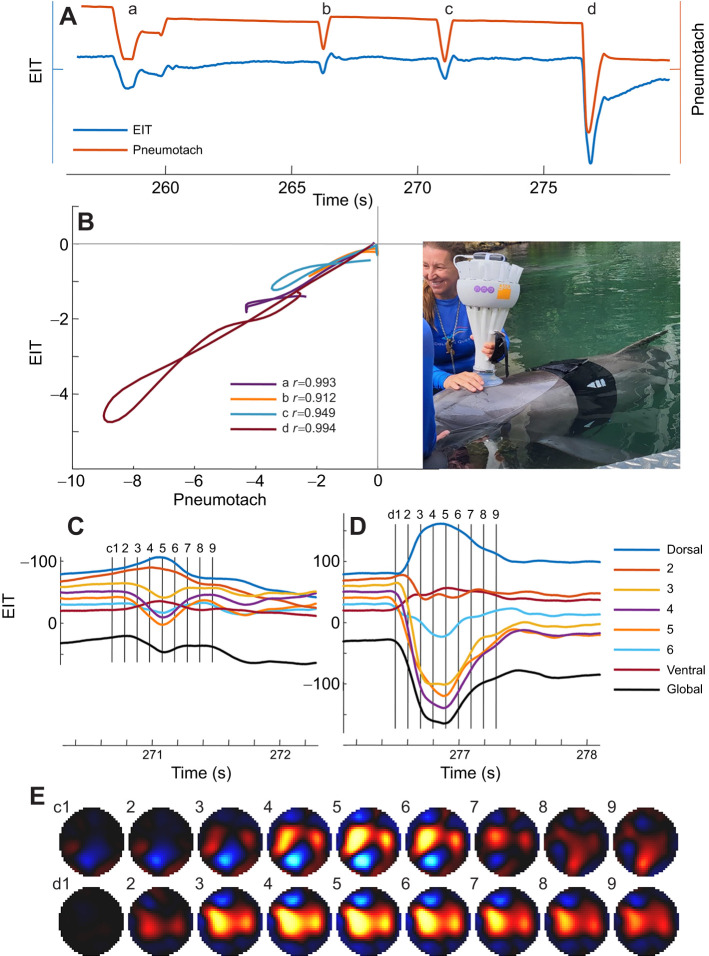
**Representative trial showing four breaths from a common bottlenose dolphin (*Tursiops truncatus*) recorded in water.** (A) Respiratory volumes from the spirometer and the EIT signal from three spontaneous (a–c) and one maximal breath (d). EIT in arbitary units and pneumotach in liters STPD. (B) Breath-by-breath slope analysis. Each breath analyzed in a different color with each breath identified by timing for breaths a–d. Image shows bottlenose dolphin wearing the EIT belt and spirometer over the blowhole. (C,D) Regional EIT signals for breaths c and d in A, respectively, representing gas distribution from dorsal to ventral regions (colored lines), as well as the global EIT signal (black line). (E) Reconstructed functional images of regional ventilation for breaths c and d, with color indicating increasing gas volume (black to blue to red to yellow). Numbers indicate corresponding timings, i.e. vertical lines in C and D.

All experiments complied with relevant ethical guidelines and regulations. EIT measurements were performed using a commercial EIT system with a custom-built electrode belt (see ‘Electrical impedance tomography’ and Figs 1 and 2), whereas respiratory flow and timing were recorded using commercial ultrasonic flow meters (see ‘Spirometry’ and Figs 1 and 2).

### Animals

A total of 21 bottlenose dolphins (*Tursiops* spp.) and eight Cape fur seals [*Arctocephalus pusillus* (Schreber 1775)] participated in this study (Table 1). The dolphin group included five common bottlenose dolphins [*Tursiops truncatus* (Montagu 1821), mean±s.d. body mass=188.8±39.5 kg] and seven Indo-Pacific bottlenose dolphins [*Tursiops aduncus* (Ehrenberg 1833), 114.7±4.8 kg] housed in managed care at Dolphin Quest Oahu (Honolulu, HI, USA) and Atlantis, The Palm (Dubai, United Arab Emirates), respectively, as well as nine wild *T. truncatus* (134.8±60.6 kg) residing in Sarasota Bay, FL, USA. The fur seal group included six individuals housed at Kolmården Wildlife Park (Sweden) and three housed at Atlantis, The Palm (85.0±36.6 kg; Table 1). Animal ID, body mass and age (known or estimated) at the time of the study are listed in Table 1.

**Table 1.** Details of individual animals participating in the study

							No. breaths/no. of trials	
Species	ID	Site	Sex	*M*_b_ (kg)	YOB	YOT	Water	Land
*Arctocephalus pusillus*	Ap4308	K	F	75	2000	2024	–	38/9
	Ap3178	K	F	44.5	2020	2024	–	1/1
	Ap1111	K	F	68.3±0.4	2013	2024/2025	–	10/3
	Ap0457	K	F	64	2013	2024	–	29/6
	Ap0110	K	F	56	2013	2024	–	13/6
	Ap0869	K	M	172.5±0.7	2011	2024/2025	–	13/8
	CFS1	ATP	F	71.5	2002	2025	5/1	–
	CFS2	ATP	M	108.5	2011	2025	6/2	–
	CFS3	ATP	M	104.5	2011	2025	–	5/3
							
*Tursiops truncatus*	9FL3	DQ	M	250.9	1985*	2024	4/2	–
	9ON6	DQ	M	186.4	1997	2024	2/1	–
	83H1	DQ	M	140.9	2008	2024	2/1	–
	6JK5	DQ	M	210.9	1994	2024	22/4	–
	01L5	DQ	M	155.0	1995	2024	17/4	–
*Tursiops aduncus*	Ta1	ATP	M	112.4	2003	2025	18/2	–
	Ta2	ATP	F	121.2	2009	2025	11/2	–
	Ta3	ATP	F	114.0	2002	2025	18/2	–
	Ta4	ATP	F	113.8	2002	2025	14/2	–
	Ta5	ATP	F	106.2	2009	2025	15/2	–
	Ta6	ATP	M	121.2	2006	2025	9/2	–
	Ta7	ATP	M	114.0	2004	2025	11/2	–
*Tursiops truncatus* (wild)	F07	SB	F	172.0	1984	2024	–	16/2
	F222	SB	M	203.0	1999	2024	–	3/1
	F261	SB	F	202.0	1975	2024	–	14/2
	F299	SB	F	104.4	2018	2024	–	3/1
	F303	SB	F	85.0	2020	2024	–	1/1
	F305	SB	F	101.0	2021	2024	–	10/2
	F330	SB	M	112.8	2019	2024	–	10/2
	F332	SB	M	98.4	2020	2024	–	16/2
	F334	SB	M	143.0	2016	2024	–	6/3

Data are shown for anonymous animal ID, site (Dolphin Quest, DQ; Kolmarden Wildlife Park, K; Atlantis, The Palm, ATP; Sarasota Bay, SB), species, sex (female, F; male, M), mean±s.d. body mass (*M*_b_), year of birth (YOB) and year of testing (YOT). Number of breaths and trials where tidal volume and Δ*Z* had an *R*²>0.85. *Estimated year of birth.

Trials with animals in managed care were conducted using operant conditioning. Participation was voluntary; animals were not restrained and could choose to disengage at any time. Dolphins and fur seals housed in managed care were desensitized to the procedures over a period of 4–6 months prior to data collection. Trials involving wild dolphins were conducted as part of routine health assessments, as previously described (Fahlman et al., 2018; Wells et al., 2004).

### Instrumentation

#### Electrical impedance tomography

A custom-built electrode belt was used for EIT measurements, consisting of 16 equally spaced electrode patches made from conductive carbon film (Haouger Electronic Technology, Shanghai) affixed in a single row onto a neoprene backing. The electrode side of the belt was positioned against the thorax, and a button connector on the opposite side linked the electrodes to the SenTec Pioneer Set (Landquart, Switzerland) for data acquisition. The system applied weak alternating currents (0.7–3.7 mA) at 150 kHz to generate tomographic images at a temporal resolution at 50 Hz. Real-time impedance waveforms and global tidal images were visualized using SenTec Monitor Software and recorded for post-processing.

To minimize current leakage and stabilize contact during in-water measurements, the electrode belt was overlaid with an additional neoprene layer and secured snugly with Velcro to reduce water accumulation between the electrodes and skin (Figs 1 and 2). Further details on the EIT reconstruction algorithms can be found elsewhere (Adler et al., 2009; Adler and Lionheart, 2006).

#### Spirometry

For dolphins, *V*_T_ was calculated from respiratory flow measurements using an ultrasonic spirometer (CetaSpiro X12; g&o embedded systems GmbH and NDD Medical Technologies), calibrated for flow rates up to 240 l s^−1^. A soft silicone interface formed a seal around the blowhole, allowing the animals to breathe freely during measurements (see fig. A1 in Fahlman et al., 2025). Respiratory flow data were transmitted via Bluetooth at 200 Hz to a laptop running custom software (Wbreath MFC Application, Version 4.0.17.0; NDD Medical Technology), which displayed real-time flow and computed integrated *V*_T_ and measured airflow.

For fur seals, an ultrasonic flowmeter (Easy-On PC; NDD Medical Technologies) was connected to a transparent polycarbonate face mask with a tight-fitting rubber gasket (Fahlman and Madigan, 2016; Fahlman et al., 2020). Data were recorded via USB using the same software as in the dolphin setup.

### Experimental procedure

#### Dolphins in managed care (in water)

A research trial began with the dolphin lining up next to the trainer, and the EIT belt was put around the chest, tightened and fastened with Velcro. Next, the dolphin floated next to the trainer, and the spirometer was placed over the blowhole (Fig. 2). The dolphin remained in this position for 2 min while the EIT and respiratory flow were measured simultaneously and continuously. Next, the spirometer was removed and the belt taken off. Each dolphin repeated the procedure later in the same day or on different days, to evaluate whether the relationship between EIT signal and respiratory flow changed.

#### Wild dolphins (on a boat deck)

Free-ranging common bottlenose dolphins residing in Sarasota Bay, Florida (27°22′40.31″N, 82°35′9.40″W), were studied during 13–24 May 2024. Juvenile and adult individuals of both sexes and varying sizes (Table 1) were sampled during routine catch-and-release health assessments conducted by the Sarasota Dolphin Research Program. Dolphins were temporarily restrained using a net, examined on a shaded, padded mat on a boat deck, and released on site following standard protocols (Wells et al., 2004).

During handling, the dolphin was gently rolled onto its side to position the EIT belt underneath. It was then rolled back onto the belt, which was pulled through and secured on the dorsal surface. A spirometer was positioned over the blowhole to record voluntary breaths for up to 10 min. Measurements were collected at the start and end of the health assessment, while the animal remained calmly positioned on the mat.

#### Cape fur seals

Eight Cape fur seals were studied either on land (sitting or lying) or while floating at the surface in water (Table 1). For all trials, the EIT belt was secured snugly around the chest, and the transparent polycarbonate mask was fitted tightly over the snout and mouth, using methods previously described (Fahlman and Madigan, 2016; Fahlman et al., 2020). In short, the mask used a rubber gasket with a central opening through which the snout fitted, effectively preventing leaks (Fahlman et al., 2020).

##### In water

During in-water trials, fur seals were either standing upright with their pectoral flippers submerged or floating horizontally with flippers resting on the pool edge with all electrodes submerged. The spirometry mask was placed securely over the snout, and animals breathed spontaneously. Up to five voluntary breaths were recorded per individual simultaneously for EIT and spirometry.

##### On land

To improve electrode contact for trials on land, each fur seal briefly entered the saltwater pool prior to testing. After exiting, excess air was removed from the fur around the chest by gently stroking the area. While the seal sat upright with flippers supporting the torso (vertical chest orientation), the EIT belt and spirometry mask were applied (Fig. 1). EIT recordings were made during a 5-min session while sitting, followed by an additional 5-min session with the seal lying prone on its chest.

Spirometry data were collected for 3–9 spontaneous breaths at the beginning or end of each session, in both postures.

### EIT processing

The EIT signal was reconstructed using a modified 3D GREIT algorithm (Adler et al., 2009; Grychtol et al., 2019) applied to an elliptical finite element model (FEM) of a marine mammal thorax. Signal processing and reconstruction were conducted using the EIDORS software suite (version 3.12; Netgen 5.3) (Adler and Lionheart, 2006). The reconstructed data generated time-dependent 2D tidal images, each representing a composite slice across the thorax formed by impedance changes between electrodes. Changes in impedance (Δ*Z*) are proportional to variations in air content within the lungs, with baseline values corresponding to end-inspiration in marine mammals.

Raw EIT data were converted into binary MATLAB files (MATLAB 9.13 R2022b) and analyzed using ibeX (v1.0) software. The resulting Δ*Z* signals were expressed in arbitrary units (AU) over time (s) and compared with corresponding volume data obtained via spirometry.

All gas volumes were standardized to STPD (standard temperature and pressure, dry) conditions (Quanjer et al., 1993). Exhaled volumes were assumed saturated at 37°C, and inhaled volumes were corrected for ambient temperature and humidity (Fahlman et al., 2015). Only complete breaths, defined as a full expiration followed by a full inspiration without any leakage (Fahlman et al., 2015; Fahlman and Madigan, 2016), were included in the analysis.

To align EIT signals with respiratory flow-derived *V*_T_, a linear regression was performed for every individual breath, producing separate calibration slopes for inhalation and exhalation. These slopes (calibration factors) were then compared within and between trials, individuals and taxa to assess consistency; we did not convert EIT data into continuous *V*_T_ traces for direct comparison. Only breaths with a strong linear relationship (*R*^2^>0.85) between *V*_T_ and Δ*Z* were retained for analysis.

This approach ensured that comparisons across breaths, sessions and subjects were based on calibrated EIT signals. It also allowed statistical analyses to meaningfully reflect variation in EIT signal performance as a volume estimator, rather than artifacts introduced by inconsistent scaling. Care was taken to differentiate between the slope used for calibration and any statistical comparisons involving group effects or inter-individual differences in breathing mechanics.

### Data processing and statistical analysis

We used generalized linear mixed-effects models (GLMMs) to evaluate how experimental and physiological factors influenced the calibration slope between exhaled or inhaled *V*_T_ and the corresponding EIT signal. Separate models were fitted for inhalation (slopein) and exhalation (slopeex) in bottlenose dolphins and Cape fur seals using the glmmTMB package in R (Brooks et al., 2017). The response variable (slope) was modeled using a gamma distribution with a log link, appropriate for continuous, positive and right-skewed data. Fixed effects included log-transformed spirometry measured *V*_T_ [log_10_(*V*_T,in_) or log_10_(*V*_T,ex_)], sex, body mass, age, testing position (sitting, lying or in water) and in some models, breath number. Interaction terms between *V*_T_ and sex were included when appropriate to test for sex-specific differences in the volume–slope relationship.

To account for repeated measures, individual animal ID (animal) and trial (trial, representing repeated tests on different days) were included as nested random effects (1|animal/trial), controlling for within-individual and within-trial variability (Littell et al., 1998). Parameter estimates, standard errors and *P*-values for fixed effects were obtained from the model output using the *summary*() function, which provides Wald *z*-tests for each coefficient. Model diagnostics were performed using the DHARMa package (https://CRAN.R-project.org/package=DHARMa), which simulates scaled residuals for GLMMs. Residuals were visually assessed using *plot*() and *hist*() functions, and their distribution was further evaluated using quantile–quantile (QQ) plots [*qqnorm*()]. Multicollinearity among fixed effects was assessed using variance inflation factors (VIF) with the *vif*() function from the car package (Fox and Weisberg, 2019).

To quantify repeatability, we used the rptR package to calculate the proportion of total variance explained by the animal:trial random effect, interpreted as the consistency of slope estimates within animals across different trials. Models were fit using linear mixed-effects models (LMMs) with a Gaussian error distribution, matching the fixed effects of the GLMMs. Confidence intervals for repeatability estimates were computed using 1000 parametric bootstraps.

## RESULTS AND DISCUSSION

This study assessed the use of EIT to estimate *V*_T_ in three marine mammal species (two dolphin species and one pinniped), measured in both aquatic and terrestrial settings. For statistical comparisons, the two dolphin species were treated as one genus (*Tursiops*) to streamline analysis. EIT measurements were validated against ultrasonic spirometry, and calibration factors (calibration slopes of the relationship between *V*_T_ and Δ*Z*) were analyzed using GLMMs. Photos of the experimental setup are shown in Figs 1 and 2, and a representative trial for a fur seal sitting on land is illustrated in Fig. 1 and a dolphin in water in Fig. 2. Across trials, a strong linear relationship was observed between EIT signal and *V*_T_, with most individual breaths exhibiting high *r*^2^ values (e.g. fur seal: mean *r*^2^_ex_=0.97±0.02, *r*^2^_in_=0.95±0.04; dolphin: mean *r*^2^_ex_=0.97±0.01, *r*^2^_in_=0.93±0.02).

### Limitations and recommendations

The number of individuals tested was relatively small, particularly for certain conditions (e.g. fur seals in water), and variability in belt placement likely introduced noise for the between-trial comparisons. Future work should focus on improving belt and electrode design to reduce motion artifacts and improve stability. Although eventual application during diving or free-swimming is an exciting possibility, current systems remain limited by cable length and waterproof electronics. Consequently, such tests were not technically feasible in the current study as at present there are no fully waterproof systems suitable for marine environments. Thus, studies on free-swimming and diving animals remain a long-term goal rather than an immediate next step. Refinement of automated breath detection and filtering algorithms will further enhance data quality.

Another challenge for future development concerns body temperature effects. Changes in impedance with temperature could influence EIT readings during active swimming or thermal variation. Accurately measuring subcutaneous temperature gradients in marine mammals is technically difficult, and temperature calibration was not attempted here. Addressing these effects will require new sensor integration and validation approaches.

Current commercial EIT systems are typically limited to sampling frequencies of ∼50 Hz, which is adequate for most human and veterinary applications. However, this limitation may be more consequential for cetaceans, given their very short breath durations and exceptionally high respiratory flows, particularly during exhalation (Fahlman et al., 2020; Fahlman et al., 2015; Kooyman and Cornell, 1981). In bottlenose dolphins, peak expiratory flows during maximal respiratory efforts have been reported to reach approximately 140–160 l s^−1^ over durations of ∼250–400 ms, with *V*_T_ approaching 90% of estimated total lung capacity (see fig. 2 in Fahlman et al., 2015). At such flow rates and time scales, a sampling frequency of 50 Hz (i.e. 20 ms temporal resolution) may be insufficient to fully resolve true peak expiratory flow, introducing a risk that peak values are temporally smoothed or underestimated in the EIT-derived signals. We therefore acknowledge that peak respiratory flows in cetaceans may be partially attenuated by the sampling limitations of current commercial EIT systems, and this represents an important methodological consideration. Future work should evaluate the magnitude of this effect, and custom-built EIT systems with higher sampling frequencies may be required to accurately capture peak respiratory dynamics in cetaceans.

Despite these constraints, our results show that EIT appears robust under controlled conditions for assessing respiratory function in marine mammals. With trial-specific calibration, it provides accurate, repeatable estimates of *V*_T_ and opens new potential avenues for both clinical and research applications. Continued development will be essential, but the potential value of EIT in advancing our understanding of marine mammal physiology is already clear.

### Tidal volume and calibration factor

Average *V*_T_ and corresponding calibration factors are summarized in Table S1. Dolphins measured in water exhibited higher *V*_T_ and calibration factors compared with those measured while beached. In contrast, calibration factors in fur seals were generally more consistent across conditions. Notably, exhalations tended to yield slightly higher calibration values than inhalations, though substantial variation was observed across trials, individuals and conditions.

The elevated *V*_T_ and calibration factors in dolphins during in-water sessions suggest more complete ventilation, whereas ventilation may be restricted when the animals are beached. These findings align with data from a larger dolphin dataset in Sarasota, where *V*_T_ decreased after dolphins had been beached for 10 min or more (Fahlman et al., 2021). In Cape fur seals, *V*_T_ was more consistent between conditions, possibly reflecting a greater adaptation to being on land compared with dolphins.

### Drivers of calibration factor variability

Using GLMMs, we tested whether calibration factors were influenced by *V*_T_ (log_10_*V*_T_), body mass, age, sex and posture (in water, sitting, laying). As shown in Table 2, log_10_*V*_T_ was the strongest and most consistent predictor of the calibration factor across all models. An interaction between sex and *V*_T_ was significant in the dolphin exhalation model, suggesting possible sex-specific differences in breathing mechanics or EIT signal propagation.

**Table 2.** Results from generalized linear mixed models (GLMMs) evaluating calibration factors to convert electrical impedance tomography (EIT) units to volume

Dependent variable	Intercept	log_10_(*V*_T_)	*M*_b_	Sex (M)	Age	Position (water)	Position (sitting)	log_10_(*V*_T_)×Sex (M)
Cetacean exhalation								
Calibration	14.1±0.4	0.91±0.41	−0.002±0.003	−0.176±0.377	0.011±0.009	0.012±0.211		−1.179±0.482
*Z*	33.4	2.22	−0.92	−0.47	1.21	0.06		−2.45
*P*-value	<0.001	0.0262	0.3574	0.642	0.228	0.954		0.0144
VIF		1.15	1.74	1.54	1.42	1.31		
Conditional model (223)	Effect	Variance	s.d.					
Trial:Animal (44)	Intercept	2.15×10^−1^	4.64×10^−1^					
Animal (21)	Intercept	3.06×10^−2^	1.75×10^−1^					
Cetacean inhalation								
Calibration	13.4±0.4	0.98±0.38	−0.001±0.003	−0.201±0.357	0.008±0.009	−0.031±0.216		−0.703±0.409
*Z*	32.9	2.61	−0.17	−0.56	0.81	−0.14		−1.72
*P*-value	<0.001	0.0090	0.861	0.573	0.418	0.885		0.085
VIF		1.15	1.90	1.60	1.61	1.36		
Conditional model (224)	Effect	Variance	s.d.					
Trial:Animal (42)	Intercept	3.33×10^−1^	5.77×10^−1^					
Animal (20)	Intercept	1.01×10^−7^	3.18×10^−4^					
Cape fur seal exhalation								
Calibration	12.1±0.5	0.87±0.18	−0.001±0.003	0.466±0.699	0.008±0.009	−0.570±0.400	0.039±0.165	0.674±0.597
*Z*	24.0	4.82	−0.17	−0.56	0.81	−1.425	0.240	1.129
*P*-value	<0.001	<0.001	0.861	0.573	0.418	0.154	0.811	0.259
VIF		1.43	3.10	3.54	1.22	1.19	1.19	
Conditional model (119)	Effect	Variance	s.d.					
Trial:Animal (38)	Intercept	1.80×10^−1^	4.24×10^−1^					
Animal (8)	Intercept	3.63×10^−2^	1.90×10^−1^					
Cape fur seal inhalation								
Calibration	11.2±0.7	0.77±0.22	0.014±0.009	−0.613±0.900	0.030±0.032	0.181±0.224	0.287±0.533	0.698±0.533
*Z*	16.3	3.52	1.537	−0.682	0.938	0.810	0.529	0.938
*P*-value	<0.001	<0.001	0.124	0.496	0.348	0.418	0.590	0.191
VIF		1.50	2.73	3.33	1.24	1.19	1.19	
Conditional model (116)	Effect	Variance	s.d.					
Trial:Animal (36)	Intercept	3.40×10^−1^	5.83×10^−1^					
Animal (8)	Intercept	7.05×10^−2^	2.65×10^−4^					

Four different models were fitted, each for exhalation or inhalation for each of the two taxa. Each model included tidal volume (*V*_T_, l STPD), body mass (*M*_b_, kg), position (on land, laying or sitting for Cape fur seals, or in water), age (years) or sex (male, M; female, F). *V*_T_ was log_10_ transformed. The models included animal and trial as random factors. The VIF function was used to test for multicollinearity among parameters. Also included are the random effects estimates from the conditional mixed-effects model using a gamma distribution (numbers in parentheses are number of observations). Variance and standard deviation are reported for the intercept across hierarchical grouping levels.

Multicollinearity was low across all models (VIFs <3.5), suggesting that explanatory variables were independently meaningful. However, body mass, age and posture had limited predictive value for the calibration factor once *V*_T_ was accounted for. This suggests that, although posture and body size may influence breathing pattern or even belt position, their impact on EIT–spirometry calibration is largely mediated through changes in *V*_T_.

### Random effects and repeatability

Variance partitioning revealed that trial nested within animal consistently explained more variation than the animal identity alone. For example, in fur seal exhalation, variance owing to trial:animal was 0.18 versus 0.036 for animal alone. This suggests that factors such as EIT belt placement, animal posture or minor shifts in behavior between sessions introduced more variability than inter-individual anatomical differences.

These patterns were confirmed through repeatability analyses (Table 3), where fur seals exhibited higher repeatability (*R*≈0.80–0.84) compared with dolphins, particularly during exhalation (*R*≈0.32). Inhalation generally showed greater repeatability than exhalation across taxa, reflecting the more forceful and variable nature of cetacean expirations. These findings emphasize the importance of calibrating each individual trial, especially for exhalation in dolphins.

**Table 3.** Breathing phase (exhalation/inhalation), repeatability (*R*), 95% confidence interval (CI) and interpretation of results

Taxa	Phase	*R*	CI	Interpretation
Cape fur seal	Exhalation	0.836	0.728–0.900	Very high consistency across trials
	Inhalation	0.799	0.670–0.879	Very high consistency
Bottlenose dolphin	Inhalation	0.641	0.485–0.742	Moderate consistency
	Exhalation	0.324	0.167–0.469	Low-to-moderate; greater trial variability

### EIT as a tool for marine mammal respiratory physiology

The strong within-trial consistency of the EIT–*V*_T_ relationship demonstrates that EIT can track respiratory volume continuously in real time. Calibration slopes (fitted separately for each breath) were highly stable within individual trials but varied slightly between trials, primarily owing to differences in belt fit, body position or electrode contact. Achieving a uniform belt fit across species and individuals remains a challenge, particularly given the large size range, from 44 kg in the smallest fur seal to 251 kg in the largest dolphin. This variation does not undermine the method, but underscores the value of trial-specific calibration, which proved both effective and practical.

Beyond volume estimation, EIT uniquely provides regional functional images of lung ventilation, revealing how air moves through different lung regions during each breath, information otherwise inaccessible in marine mammals. Fig. 2 illustrates this capability: synchronized spirometry and EIT traces from a dolphin show calibration relationships, regional EIT waveforms, and sequential functional images in which increasing gas volume is represented by color progression from black to yellow. These spatiotemporal maps offer new insight into the timing and distribution of ventilation, highlighting the ability of EIT to resolve complex respiratory mechanics in dive-adapted species.

Our study demonstrates that EIT can provide accurate, real-time imaging of ventilation patterns in marine mammals in both air and seawater, something previously thought technically infeasible owing to electrical interference in conductive environments. By validating this capability, we have bridged a key technological gap between clinical EIT and physiology in marine mammals, showing that it can operate reliably with seawater contact and dynamic thoracic geometry.

This breakthrough establishes a foundation for future waterproof, wireless EIT systems capable of recording during static dives or controlled submersion. Although fully free-swimming applications remain aspirational, this work proves that the core principle, quantitative EIT imaging of a submerged marine mammal, is feasible, dependent on significant hardware development.

### Conclusions

Our findings demonstrate that EIT reliably tracks *V*_T_ in both dolphins and fur seals, with calibration factors showing remarkable within-trial stability across environments and breathing phases. Repeatability was particularly strong in fur seals and during inhalation, suggesting that certain species- and phase-specific factors may influence signal consistency. Together, these results support EIT as a reliable and versatile research tool under controlled conditions. With continued technical refinement, it may eventually contribute to studies of lung mechanics and respiratory health in marine mammals exposed to the challenges of diving. Although present systems are restricted to surface or static dives, our demonstration that EIT functions accurately in seawater establishes a crucial technological proof-of-concept. This milestone transforms underwater respiratory imaging from an aspiration into an achievable near-term goal, providing a pathway to directly observe lung mechanics during diving.

## Supplementary Material

10.1242/jexbio.251412_sup1 Supplementary information
