# Supplementary material for: Using electrical impedance tomography to estimate tidal volume in bottlenose dolphins and cape fur seals in seawater and on land
Source: J Exp Biol. 2026 Feb 26;229(4):jeb251412. doi: 10.1242/jeb.251412 (PMC12967136; doi:10.1242/jeb.251412)
Supplement: Supplementary information [file jexbio-229-251412-s1.pdf]

Table S1. Average (± s.d.) tidal volume (VT) and calibration factor (CAL) for exhalation (Ex) and inhalation (In) for bottlenose dolphins (CET) and Cape fur seals (CFS) positioned (position) in water (W) or on land (L). Superscripted numbers for tidal volume are the number of animals measured in each position.

Variable	VTex		VTin		CALex		CALin	
Position	W	L	W	L	W	L	W	L
CET	4.6±1.9^{12}	3.4±1.2^{9}	4.2±2.1^{11}	3.4±1.4^{9}	1335812±832475	951917±583602	505586±273379	674909±293196
CFS	2.3±1.0^{3}	2.0±1.3^{5}	2.4±1.3^{3}	2.0±1.4^{5}	575865±463337	631892±564566	783123±547360	748158±813397

Journal of Experimental Biology • Supplementary information
